# An Erythropoietin-Independent Mechanism of Erythrocytic Precursor Proliferation Underlies Hypoxia Tolerance in Sea Nomads

**DOI:** 10.3389/fphys.2021.760851

**Published:** 2022-01-27

**Authors:** Melissa Ilardo, Maria C. Ferreira dos Santos, Niels Grote Beverborg, Malini Rajan, M. Abdullah Said, Niek Verweij, Pim Van Der Harst, Peter Van Der Meer, Elizabeth A. Leibold

**Affiliations:** ^1^Maze Therapeutics, San Francisco, CA, United States; ^2^Department of Internal Medicine, Division of Hematology and Hematologic Malignancies, The University of Utah, Salt Lake City, UT, United States; ^3^University Medical Center Groningen, Groningen, Netherlands

**Keywords:** hypoxia, red blood cell production, adaptation, evolution, spleen

## Abstract

The Bajau Sea Nomads were recently demonstrated to have evolved larger spleens as an adaptation to millennia of a marine foraging lifestyle. The large-spleen phenotype appears to derive from increases in thyroid hormone (TH) production as a result of reduced expression of phosphodiesterase 10A (PDE10A), though the exact mechanism remains unknown. Through pharmacological inhibition of PDE10A using the selective inhibitor MP-10 in mice, we were able to mimic the Bajau adaptation and show that treated mice had significantly larger spleens than control animals. This difference appears connected to an excess of early stage erythrocytes and an apparent increase in red blood cell (RBC) precursor proliferation in response to increased TH. However, we determined that the stimulation of RBC production in the mouse model *via* TH is Erythropoietin (EPO)-independent, unlike in the altitude (chronic hypoxemia) response. We confirmed this using human GWAS data; although the Bajau PDE10A variants are significantly associated with increased TH levels and RBC count, they are not associated with EPO levels, nor are other strongly thyroid-associated SNPs. We therefore suggest that an EPO-independent mechanism of stimulating RBC precursor proliferation *via* TH upregulation underlies the increase in spleen size observed in Sea Nomad populations.

## Introduction

Thyroid hormones affect a wide variety of metabolic and physiological processes, and were recently linked to increased spleen size as an apparent adaptation to breath-hold diving (Ilardo et al., 2018). However, the spleen-thyroid relationship is not new; as early as 1,927 hyperthyroidism was reported in medical contexts to be associated with large spleen size (Baldridge and Peterson, 1927), and at one time splenomegaly was used as a diagnostic tool. The exact mechanisms by which thyroid hormones may affect spleen size are yet unknown. Here, we report an erythropoietin-independent mechanism of red blood cell (RBC) production as a consequence of thyroid hormone stimulation *via* the pharmacological inactivation of Phosphodiesterase 10A (PDE10A).

Phosphodiesterase 10A is a phosphodiesterase capable of hydrolyzing both cAMP and cGMP, thus regulating cyclic nucleotide signaling (Fujishige et al., 1999). It was recently discovered that PDE10A expression has been genetically reduced in a population called the Bajau as a consequence of natural selection acting on the ability to sustain repeated bouts of apneic diving (Ilardo et al., 2018). This reduction in expression has resulted in two apparent phenotypes, the first of which is increased thyroid hormone levels. Although thyroid hormones have not been directly measured in the Bajau population, a genome wide association scan (GWAS) for Thyroid Stimulating Hormone (TSH) levels (a proxy for thyroid hormones with high sensitivity), revealed a strong association between SNPs in PDE10A as well as in related phosphodiesterase PDE8B, which encodes a high affinity cAMP-specific phosphodiesterase (Arnaud-Lopez et al., 2008; Soto-Pedre et al., 2017). The second phenotype associated with reduced PDE10A expression is splenomegaly. Spleen size has been proposed to affect dive capacity owing to the spleen’s role in the mammalian dive reflex, in which it contracts during diving in order to expel a bolus of oxygenated RBCs thus expanding available oxygen (Thornton et al., 2001). The large spleen phenotype observed in the Bajau has been proposed to enhance their dive capacity, though the mechanisms underlying the adaptations are unknown.

Here we use a pharmacological approach to inhibit PDE10A in mice in order to investigate the molecular mechanisms by which thyroid hormones are connected to an increase in spleen size in the Bajau. We utilized the highly selective PDE10A inhibitor MP-TRA 10 (MP-10), which has previously been shown to potentiate thermogenesis, suggesting a thermoregulatory role (Hankir et al., 2016). Through daily injections, we were able to mimic the genetic effect of the Bajau adaptation in mice. We subsequently observed a significant increase in spleen size compared to control animals, as well as a corresponding increase in RBC count originating in an excess proliferation of erythrocytic precursors in the spleen, which in mice, unlike in humans, is a site of erythropoiesis. Further, we demonstrate that this stimulation of proliferation is likely erythropoietin (EPO) independent, suggesting a direct role of thyroid hormones on erythrocytic precursor development. These data suggest the benefits of Bajau PDE10A adaptation are two-fold; first, the increased RBC count would increase oxygen capacity allowing for longer dives on a single breath, and secondly the corresponding increase in spleen size creates a larger reservoir in which the oxygenated red blood cells may be stored.

## Results

**FIGURE 1:**
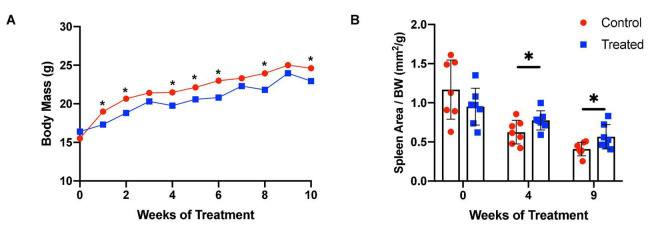
MP-10-treated mice have lower body weights and larger spleens than control animals *in vivo*. **(A)** MP-10-treated (10 mg/kg) mice demonstrate consistently lower body weights than control mice, with a significantly lower change in body weight over the period (*p* = 0.029) (*n* = 14). **(B)** Mice receiving MP-10 (10 mg/kg) had larger spleen area as measured using a small animal ultrasound machine (*n* = 14). All comparisons were performed using an unpaired Student’s *t*-test and are plotted with SD error bars. ^∗^*p* < 0.05.

**FIGURE 2:**
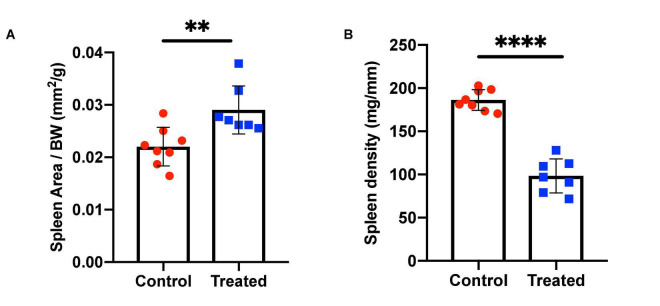
Spleen area and density in control and MP-10 treated mice. **(A)** Mice treated with a short course of MP-10 (1 week, 30 mg/kg) displayed larger spleens (*p* = 0.0038) that were also **(B)** less dense (*p* < 0.0001) (*n* = 15). All comparisons were performed using an unpaired Student’s *t*-test and are plotted with SD error bars. ^∗∗^*p* ≤ 0.01 and ^∗∗∗∗^*p* ≤ 0.0001.

**FIGURE 3:**
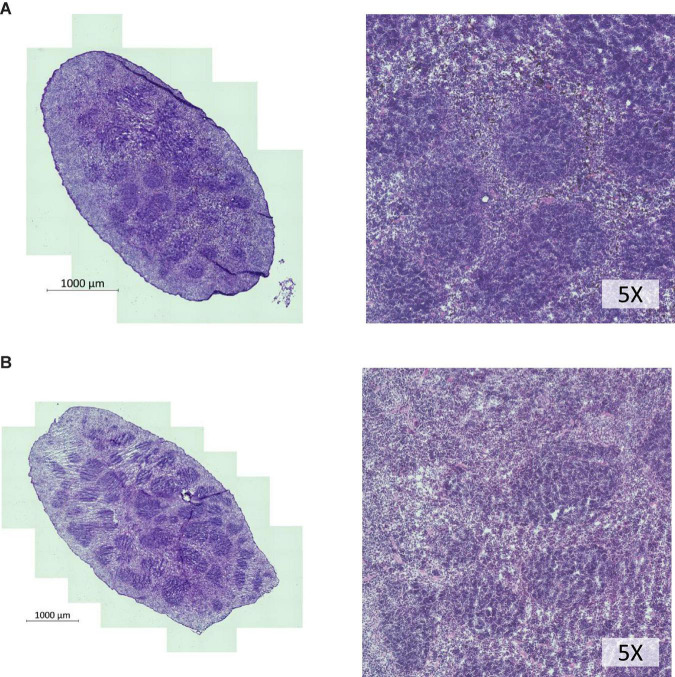
H&E staining of spleen cross sections of whole spleens shows lower cellular density in spleens of MP-10 treated mice. **(A)** Control mice and **(B)** MP-10 treated mice from the short-term study (30 mg/kg). Representative data from *n* = 15 mice per group.

**FIGURE 4:**
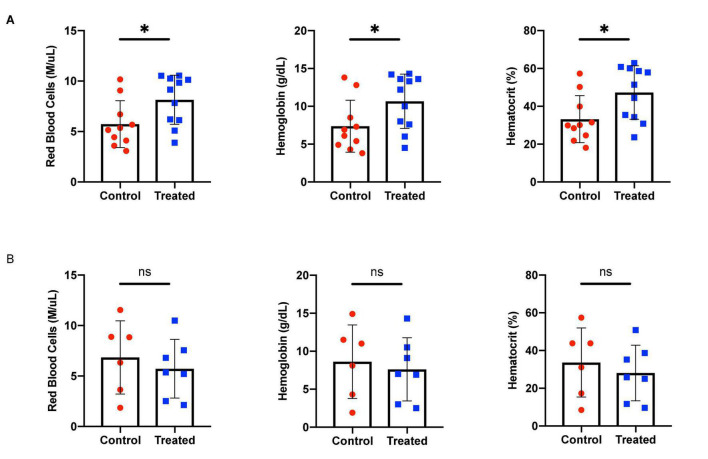
Hematological effects of short- and long-term phosphodiesterase 10A (PDE10A) inhibition. **(A)** After 1 week of treatment, mice receiving MP-10 (10 mg/kg) displayed significantly higher red blood cell (RBC) counts (*p* = 0.0317), an effect also confirmed in humans carrying the Bajau genetic variant (Table 1). Treated mice were also found to have significantly higher hematocrit (*p* = 0.0259) and hemoglobin (*p* = 0.0458) than control animals. **(B)** After 9 weeks of treatment with MP-10 (30 mg/kg), there are no apparent hematological differences between treated and control animals. ^∗^*p* ≤ 0.05.

We chose to divide our pharmacological model into two studies conducted over different time courses; a long-term study with lower dosage to represent chronic, mild inhibition of PDE10A, and a short-term study with higher dosage to represent more acute inhibition. For both studies there is precedent in the literature (Hankir et al., 2016), and both provide different perspectives to inform us of the mechanisms of response to PDE10A inhibition. While the long-term study may more closely represent inhibition in humans with the Bajau variant in PDE10A, the short term study may more closely resemble a knockout model. For our long-term study, we injected 4-week old male mice daily *i.p.* with a low (10 mg/kg) dosage of MP-10 over a period of 9 weeks. We observed lower body weights in treated mice compared to control mice (Figure 1). These differences are consistent with previous observations in mouse studies that administered similar dosages of MP-10 (Hankir et al., 2016), and we believe are likely a consequence of increased basal metabolic rate (BMR) resulting from thyroid hormone stimulation. Following 0, 4, and 9 weeks of treatment, we measured spleen size *in vivo* using a small animal ultrasound. We found that after 4 and 9 weeks of treatment the treated mice had larger spleens *in vivo* than the control animals (*p* = 0.0634, and *p* = 0.0374, respectively), consistent with the large spleen phenotype observed in the Bajau (Figure 1; Ilardo et al., 2018). We repeated the experiment at a higher dosage (30 mg/kg) for a shorter time course of 1 week. Following 1 week of treatment, treated mice had gained significantly less weight than controls (*p* = 0.0019) (Figure 2). Through *ex vivo* spleen measurements of length and width, we determined that the treated mice again had significantly larger spleens than the control mice (*p* = 0.0059, Figure 2). However, the spleens of treated mice were also significantly less dense than those of the control animals (*p* < 0.0001, Figure 2), as calculated by a ratio of measured splenic area and splenic mass. Spleen sections were stained with H&E, and differences were clearly visible and appear to arise from increased distance between cells (Figure 3). We also observed that the treated mice had higher RBC count, hematocrit (Hct), and hemoglobin (Hgb) (Figure 4). While our hematological values were consistently low across all animals, they were within the normal range for young animals (Wozniak et al., 2021). These findings are consistent with the spleen becoming stretched, and thus less dense, suggesting that they may serve as a reservoir for a greater number of red blood cells. It is, however, difficult to say whether this increase is indeed due to a greater RBC mass because of the potentially confounding effect of changes in blood volume. Such a determination would require chromium labeling or CO rebreathing to accurately determine RBC mass. Using GWAS data from the UK Biobank (accessed using the Global Biobank Engine, Stanford, CA,^1^ December 2019), we identified consistent results in humans of European ancestry. Humans carrying the Bajau variant at previously identified SNPs known to influence PDE10A expression were also found to have significantly higher RBC count than those carrying the ancestral variant (Table 1). We have not, however, confirmed this association in the Bajau population.

**TABLE 1:** Phosphodiesterase 10A (PDE10A) SNPs are associated with increased red blood cell (RBC) count but not erythropoietin (EPO).

SNP	Chr	Gene	RBC count assoc	EPO assoc
rs3008049	6	PDE10A	2.658 × 10^–3^	0.4091598959
rs3008050	6	PDE10A	7.255 × 10^–3^	0.226484351
rs3008052	6	PDE10A	− ^a^	0.364422382
rs2983527	6	PDE10A	1.128 × 10^–2^	0.2426864261

*Red blood cell count (from UK Biobank) and EPO (from the PREVEND cohort) association at SNPs of interest. The Bonferroni corrected thresholds for significance are 0.0167 (for RBC), 0.0125 (for EPO).*

*^a^This SNP is not available in the UK Biobank data.*

**FIGURE 5:**
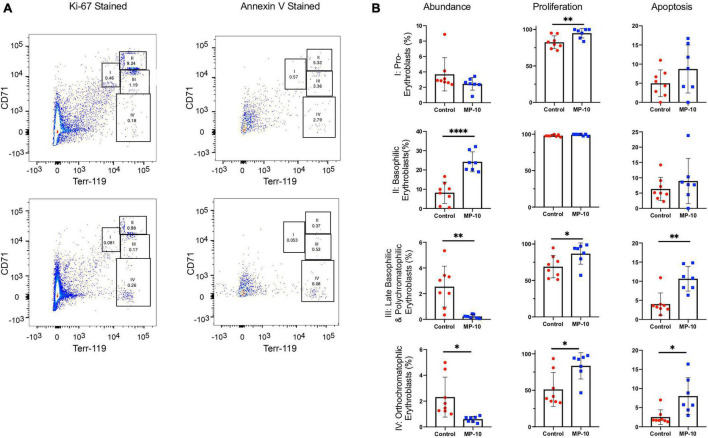
Flow cytometry data show differences in cell precursor populations between control and MP-10 treated mice. **(A)** Representative flow cytometry data of splenic cells from a control mouse and MP-10 (30 mg/kg) treated mouse stained with Ki-67, an indication of proliferation and AnnexinV, an apoptosis marker. The four different erythrocytic populations were defined according to Socolovsky et al. (2001): I: pro-erythroblasts; II: basophilic erythroblasts; III: late basophilic erythroblasts; IV: orthochromatophillic erythroblasts. **(B)** Analyzed flow cytometry data demonstrate MP-10 (30 mg/kg) treated animals have significantly increased proliferation at nearly every stage of development. Comparison of splenic cellular composition between control and MP-10 treated mice demonstrate that treated mice have an increase in proliferation in nearly every precursor population, however proportionally lower abundance of late stage precursors due to increased apoptosis. Results are summarized in Table 2. ^∗^*p* ≤ 0.05, ^∗∗^*p* ≤ 0.01, and ^∗∗∗∗^*p* ≤ 0.0001.

Through flow cytometry, we were able to attribute the difference in hematological measures to differences in erythrocytic precursor populations (Figure 5). We found a significant increase in proliferation at nearly every stage of cell proliferation (Figure 5 and Table 2 for a summary of *p*-values), however late stages (III and IV, representing late basophilic erythroblasts and orthochromatophillic erythroblasts, respectively) were also found to have a significant increase in apoptosis, resulting in an overall lower proportional abundance of cells in these stages. This balancing effect may be mediated by thyroid hormones and serve to ensure a normal number of reticulocytes is released.

**TABLE 2:** Flow cytometry results summary.

Precursor population/gate	Relative abundance	Proliferation	Apoptosis
Pro-erythroblasts (I)	0.1773	0.0087	0.1718
Basophilic erythroblasts (II)	<0.0001	0.0692	0.3967
Late basophilic & polychromatophilic erythroblasts (III)	0.0020	0.0374	0.0011
Orthochromatophillic erythroblasts (IV)	0.0127	0.0107	0.0102

*P-values for flow results, blue or red values indicate the relative proportion of cells under a given designation is significantly higher in treated or control mice, respectively.*

In order to investigate whether the underlying cause of increased RBC precursor proliferation could be thyroid hormone induced stimulation of erythropoietin (*Epo*), we used qPCR to measure *Epo* mRNA in kidney. We found no difference in *Epo* expression between control and MP-10 treated mice (*p* = 0.48). Using GWAS data from Prevention of REnal and Vascular ENd (PREVEND), we confirmed this result in humans of European ancestry. In spite of a clear, significant association with thyroid hormone levels previously reported in Ilardo et al. (2018), there is no association between the Bajau PDE10A SNPs and *Epo* levels (Table 1). We investigated additional SNPs in other genes known to be strongly and significantly associated with thyroid hormone levels in European populations, none of which were found to be associated with Epo levels (Table 3). We have yet to confirm these results in Bajau individuals.

**TABLE 3:** Thyroid Stimulating Hormone (TSH) association does not correlate with *Epo* association.

SNP	Chr	Gene	TSH assoc.	EPO assoc
rs2046045	5	PDE8B	1.85 × 10^–17^	0.7699698027
rs6885099	5	PDE8B	1.95 × 10^–56^	0.723791259
rs1382879	5	PDE8B	7.16 × 10^–18^	0.5133130162

*Thyroid Stimulating Hormone and EPO association values for three significantly thyroid-associated SNPs are found in another phosphodiesterase (PDE8B) known to be associated with thyroid hormones (Panicker et al., 2008; Malinowski et al., 2014).*

## Discussion

Through a pharmacological approach in mice, we were able to simulate the Bajau large spleen adaptation to breath hold diving. We administered daily injections of the selective PDE10A inhibitor MP-10 daily *via ip* injection at low (to measure chronic inhibition) and high (to measure acute inhibition) dosages for 9 weeks and 1 week, respectively. Using a small-animal ultrasound, we determined that the treated mice had significantly larger spleens *in vivo* than the control animals, confirming the phenotype observed in the Bajau. This phenotype was replicable *ex vivo*, however the spleens of treated mice were significantly less dense than those of the control animals. We attribute this difference in splenic density to a stretching of the tissue to accommodate a greater quantity of RBCs, apparent through hematological testing.

We attribute the increase in RBC count to an observed significant excess of early stage erythrocytic precursors in treated mice, which has been previously identified as a response to thyroid hormone stimulation (Angelin-Duclos et al., 2005). Thyroid hormones are known to be required for terminal erythroid differentiation, however the exact molecular mechanisms underlying thyroid hormone function on erythropoiesis are unknown (Gao et al., 2017). In agreement with our results, thyroid hormones have been previously demonstrated to directly affect proliferation of erythroid progenitors (Golde et al., 1977; Popovic et al., 1977; Dainiak et al., 1978; van Gucht et al., 2017). This may be attributable to higher levels of TH nuclear receptors α (TRα) in early progenitors compared to late stage erythroblasts, as has been previously demonstrated to underlie variable responses to thyroid hormones at different stages of development (Gao et al., 2017).

We also note a significant increase in apoptosis of late-stage erythrocytic precursors in the spleens of treated mice, a previously characterized response to splenomegaly (Ilesanmi, 2010). We suggest that thyroid hormones mediate an apoptotic feedback mechanism that, in response to increased erythrocytic precursor stimulation, controls the number of reticulocytes that are released and is preventative of red blood cell excess. However, the apparent apoptotic mechanism proves insufficient in the short term to overcome the excess production of early precursors; after 1 week of treatment, the experimental mice displayed pronounced polycythemia (Figure 4). It appears that over longer periods, in the case of our study a 9-week administration of MP-10, the feedback returns hematological values to normal levels (Figure 4). This could be a limitation of the pharmacological model and the result of slight inconsistencies in delivery of the low dosage of MP-10 over the 9 weeks. A knockout model would provide more consistent inhibition of PDE10A and more reliable hematological values. It could, however, be truly representative of what occurs in humans and therefore the reason hyperthyroidism has not yet been consistently linked to polycythemia.

A reduction in RBC count, and a corresponding reduction in EPO has been previously reported in hypothyroid patients, however the reduction in EPO was thought to be associated with reduced oxygen requirement due to diminished BMR (Das et al., 1975). In fact, it has been suggested that thyroid hormones only induce EPO production under hypoxic conditions (Fandrey et al., 1994). *In vitro* studies have shown that thyroid hormones can stimulate erythropoiesis directly in systems where hormonal effects on EPO production are eliminated (Malgor et al., 1975; Golde et al., 1977), thus suggesting an EPO independent mechanism, consistent with our findings which showed no difference in *Epo* expression between treated and control animals. It may be that slight differences in expression are obscured by the nature of the measurement. Unfortunately, we were not able to measure TH or EPO in the animals directly. In addition, it is important to note that while *Gapdh* is frequently used for normalization in other TH studies, it is a HIF-dependent gene and as such may change in expression along with our gene of interest.

It is important to note that the adult human spleen is not a site of erythropoiesis as it is in mice. Because of this, observed cell population differences in the mouse spleen should be considered analogous to bone marrow in humans (Brodsky et al., 1966; Bresnick et al., 2018). In addition, erythroblastic islands, which are essential for the maturation of erythroblasts that are fated to enucleate, have been demonstrated *in vivo* in fetal splenic red pulp (Manwani and Bieker, 2008). Therefore, the observations in this study may be extrapolated to human fetal development and the observed changes in spleen size may occur developmentally.

## Materials and Methods

### Mice

For this study, we used wild type C57BL/6J mice. Experiments were performed on weaned animals beginning at 3 and 4 weeks for the short- and long- term studies, respectively. These ages were chosen because most murine spleen development occurs by 3 weeks (Hankir et al., 2016). While the neonatal mouse spleen is a hematopoietic site, by the time of full development the spleen’s role is limited to erythropoiesis (Wolber et al., 2002; Angelin-Duclos et al., 2005).

We only included males because of well of documented, significant differences in free T4 between males and females in various recombinant inbred (RI) strains of mice which create a sex-dependent thyroid hormone signal (McLachlan et al., 2014). To inhibit PDE10A, we used the highly selective PDE10A inhibitor PF-2545920 hydrochloride (MP-10) from Sigma-Aldrich dissolved in DMSO and diluted in 40% 2-Hydroxypropyl-beta-cyclodextrin (HPβCD). In the long-term study, animals were injected daily with either 10 mg/kg of MP-10 or an equivalent volume of vehicle intraperitoneally (*i.p.*) for 9 weeks and were subsequently sacrificed. For the short-term study, animals were injected daily with 30 mg/kg MP-10 or an equivalent volume of vehicle *i.p.* for 1 week, after which they were sacrificed. In both the long- and short-term study, the final injection was performed 2 h before the animals were sacrificed.

### Ultrasound Studies

Images were acquired with B-mode, and color Doppler modes using a Vevo 2100 high frequency ultrasound machine (VisualSonics) and a MS550D 22–55 MHz probe. Animals were sedated with isoflurane and scan time was approximately 20 min to identify and image the spleen. Spleen area was calculated from 2-dimensional images using the VisualSonics software.

### Flow Cytometry

Whole spleens were collected, washed in RPMI and PBS and passed through 100 and 70 μM mesh to release cell suspensions. Single cell suspensions obtained from spleens were analyzed with the following antibodies: B220-APC, TER119-PE, CD71-FITC and Annexin V PerCP-eFluor710 (eBioscience). Cells were analyzed on a BD LSRFortessa machine (Becton Dickinson) using FlowJo software. The four different erythrocytic populations were defined according to Socolovsky et al. (2001): I: Ter119*^med^*CD71*^high^* (pro-erythroblasts); II: Ter119*^high^*CD71*^high^* (basophilic erythroblasts); III: Ter119*^high^*CD71*^med^* (late basophilic erythroblasts); and IV: Ter119*^high^*CD71*^low^* (orthochromatophillic erythroblasts). For compensation, single positive labeled cells and OneComp eBeads (Becton Dickinson) were used. Ki-67 PerCP-eFluor710, the intracellular staining to identify proliferating cells was performed according to the Intracellular Fixation & Permeabilization Buffer Set protocol (eBioscience).

### Erythropoietin qPCR

For indicated conditions and time-points, tissues were collected and minced followed by RNA extraction using TRIzol Plus RNA Purification Kit (Invitrogen Cat#: 12183555). cDNA synthesis was performed using qScript XLT cDNA SuperMix (QuantaBio Cat# 95048-025). After reverse transcription, qPCR was performed using TaqMan Gene Expression Assays (Applied Biosystems) and analyzed using an Applied Biosystems 7900 HT qPCR instrument. The cycle threshold (Ct) value for each transcript was normalized to *Gapdh*. The comparative Ct method was used to quantify transcript abundance. TaqMan Assays used were; Epo: Mm01202755_m1, Gapdh: Mm99999915_g1 Gapdh.

### Histology

Spleens samples were submitted to histology for sectioning and Hematoloxin and Eosin staining. The frozen tissue was sliced at a thickness of 5 microns. Prepared slides were imaged with the Axioslide scanner.

### Hematology

Blood was collected from the submandibular vein 24 h before animals were sacrificed. Complete blood counts were conducted using a Drew Scientific Hemavet 950FS with manufacturer mouse settings.

### Prevention of REnal and Vascular ENd-Stage Disease Study

Participants from the PREVEND study were used for this study. PREVEND has been used for the association studies with EPO levels, for details on the study protocol see Hillege et al. (2001); Grote Beverborg et al. (2015). In brief, PREVEND was designed to prospectively investigate the natural course of urinary albumin excretion (UAE) and its relationship with renal and cardiovascular disease in a large cohort drawn from the general population. For the current analyses, we used data from the second survey between 2001 and 2003. Serum EPO levels were measured using the IMMULITE EPO assay (DPC, Los Angeles, CA, United States), and a luminometer measured the amount of serum EPO using chemiluminescence. We used 2,691 randomly selected non-anemic individuals, to avoid confounding variables. Analysis was performed on residuals of EPO levels after adjustment for age, gender and the first 10 principal components using an additive genetic model in SNPTEST v2.4.1.

## Data Availability Statement

The original contributions presented in the study are included in the article/supplementary material, further inquiries can be directed to the corresponding author.

## Ethics Statement

The animal study was reviewed and approved by the University of Utah IACUC.

## Author Contributions

MI devised and performed the experiments and wrote the manuscript. MS assisted with the designing and implementing all the experiments as well as data interpretation. NG, NV, PiV, and PeV generated, analyzed, and interpreted the EPO GWAS data. MR assisted with the experiments. EL assisted in the experimental design. All authors reviewed the manuscript.

## Conflict of Interest

MI was employed by the company Maze Therapeutics. The remaining authors declare that the research was conducted in the absence of any commercial or financial relationships that could be construed as a potential conflict of interest.

## Publisher’s Note

All claims expressed in this article are solely those of the authors and do not necessarily represent those of their affiliated organizations, or those of the publisher, the editors and the reviewers. Any product that may be evaluated in this article, or claim that may be made by its manufacturer, is not guaranteed or endorsed by the publisher.

## Footnotes

1
https://biobankengine.stanford.edu/
